# The effectiveness of nab-paclitaxel plus gemcitabine and gemcitabine monotherapy in first-line metastatic pancreatic cancer treatment: A real-world evidence

**DOI:** 10.1097/MD.0000000000030566

**Published:** 2022-09-30

**Authors:** Juraj Prejac, Dora Tomek Hamzić, Nikša Librenjak, Irma Goršić, Domina Kekez, Stjepko Pleština

**Affiliations:** a University Hospital Centre Zagreb, Department of Oncology, Zagreb, Croatia; b University of Zagreb, School of Dental Medicine, Zagreb, Croatia; c University of Zagreb, School of Medicine, Zagreb, Croatia.

**Keywords:** first-line treatment, gemcitabine, nab-paclitaxel, pancreatic cancer

## Abstract

Pancreatic cancer is one of the most lethal malignancies with a rise in mortality rates. FOLFIRINOX and nab-paclitaxel plus gemcitabine demonstrated a survival benefit compared to gemcitabine alone. Both protocols are now considered the standard of first-line treatment with no significant difference between them, primarily based on observational studies. Although new therapeutic options have emerged recently, the prognosis remains poor. We conducted a retrospective single-center study on 139 patients treated for metastatic pancreatic adenocarcinoma (mPDAC) with gemcitabine monotherapy (Gem) or nab-paclitaxel + gemcitabine (Nab-P/Gem) in the first line. The aim of our study was to evaluate the effectiveness in terms of overall survival (OS) and progression-free survival (PFS) as well as the influence of patient and disease characteristics on outcomes. Nab-P/Gem resulted in OS of 13.87 months compared to 8.5 months in patients receiving Gem. The same trend was achieved in PFS, 5.37 versus 2.80 months, respectively, but without reaching statistical significance. Furthermore, the 6-month survival in the Nab-P/Gem group was also higher, 78.1% versus 47.8%. In terms of survival, the group of elderly patients, patients of poorer performance, with higher metastatic burden and liver involvement, benefited the most from combination therapy. In our analysis ECOG performance status (p.s.), previous primary tumor surgery, and liver involvement were found to be independent prognostic factors. The addition of nab-paclitaxel to gemcitabine resulted in a significant improvement in the OS of patients with mPDAC. Subgroup analysis demonstrated that patients with some unfavorable prognostic factors benefited the most.

## 1. Introduction

Pancreatic cancer is one of the most lethal malignancies worldwide based on GLOBOCAN 2020 estimates.^[1]^ It is ranked as the 13th most common cancer in the world and 7th cause of cancer-related death.^[1]^ Similarly to global estimates, it is the 8th most common cancer in Croatia.^[2]^ Its dismal prognosis is due to the majority of patients having advanced or metastatic disease at the time of diagnosis^[3]^ accompanied by a 5-year survival rate of <9%.^[4]^ Despite all new improvements, such as combination with nab-paclitaxel or FOLFIRINOX protocol in the first line or nanoliposomal irinotecan in the second line, treatment is still insufficient.

For the past 2 decades, gemcitabine alone was the only standard treatment for locally advanced and metastatic pancreatic ductal adenocarcinoma (mPDAC) with survival of <6 months.^[5]^ Based on trial results published in 2011 and 2013, FOLFIRINOX and nab-paclitaxel plus gemcitabine, became therapies of choice in the first-line setting.^[6,7]^ Today, both regimens are considered standard of care in the first-line setting with no head-to-head comparison.

New first-line treatment options resulted in the increase in the proportion of patients receiving second and third-line therapy.^[8]^ Better treatment options resulted in higher probability of response rate, overall survival (OS), and longer preservation of quality of life.^[6,7]^ Introduction of new therapies, as such, resulted in higher number of patients receiving second and third-line treatments.^[8,9]^ For example, Kieler et al^[9]^ studied the impact of new chemotherapy regimens on survival of mPDAC patients and found significantly increased mOS for patients who started systemic treatment in the later time period, after the introduction of novel therapies (calculated both from the beginning of the first as well as the second line). However, optimal first-line treatment is still challenging.

MPACT (a randomized phase III study of weekly ABI-007 plus gemcitabine versus gemcitabine alone in patients with metastatic adenocarcinoma of the pancreas) was a randomized, phase III trial demonstrating superiority of nab-paclitaxel added to gemcitabine versus gemcitabine alone. The median OS was 8.5 months in the combination group compared to 6.7 months in the control group. In general, patients with more advanced disease poorer performance status (p.s) ≥1 measured according to Eastern Cooperative Oncology Group (ECOG p.s. scale), high carbohydrate antigen 19-9, presence of liver metastasis or more than 3 sites of metastatic disease had the greatest reduction of risk of death.^[7]^ Proportions of grade ≥3 adverse events (adverse events which are severe or medically significant but not immediately life-threatening and hospitalization or invasive intervention is required according to Common Terminology Criteria for Adverse Events v.5)^[10]^ were higher in the nab-paclitaxel group with myelosuppression, asthenia, and peripheral neuropathy being the most common.^[11]^ The real-world evidence on the efficacy of addition of nab-paclitaxel to gemcitabine is mostly limited to short follow-up with no specific patient and disease characteristic analysis.^[12–17]^ A recently published meta-analysis including the total of 26 studies reported a median OS ranging from 6.9 to 24.7 months across 19 studies and overall response rate of 31.6% in 24 studies.^[12]^ In the aforementioned article, the median number of patients was less than 100. Considering the limited data and on a relatively small number of patients in a real-world setting, additional evidence on effectiveness of chemotherapy for advanced pancreatic cancer is needed. Therefore, we aimed to evaluate survival outcomes for patients with mPDAC treated with nab-paclitaxel and gemcitabine (Nab-P/Gem) or gemcitabine alone (Gem) in University Hospital Centre Zagreb. Furthermore, the emphasis was also placed on the analysis of available potential prognostic and predictive factors related to patient and disease characteristics.

## 2. Methods

In this retrospective, observational, single-center study conducted in the UHC Zagreb in Zagreb, Croatia, data were retrieved from medical records on 139 patients who began first-line treatment for mPDAC between January 1st, 2015 and January 1st, 2020. Study was approved by the institutional ethics committee. All the eligible patients were at least 18 years old, had histologically or cytologically confirmed stage 4 disease when the treatment was initiated, and completed the first-line therapy with Nab-P/Gem or Gem by December 1st, 2020. Data from medical records for each patient were available from the time of diagnosis of pancreatic cancer until death or the most recent follow-up visit in our center.

Treatment consisted of Nab-P/Gem or Gem. The Nab-P/Gem group included 64 patients (34 men, 30 women) and the Gem group included 75 patients (42 men, 33 women). The therapy regimen was chosen by the attending physician and depended on the patient’s overall health (ECOG p.s.) and preferences. Nab-paclitaxel reimbursement is fully covered by Croatia’s public funds, The Croatian Health Insurance Fund, since the end of 2016 for patients with ECOG p.s. 0 or 1 with metastatic pancreatic cancer. Before that date, patients were treated with gemcitabine alone. Patients with ECOG p.s. >1 were excluded from the study as they were not table to receive Nab-P. And, only patients with metastatic disease, as opposed to locally advanced pancreatic cancer, were included. Patients treated for the non-metastatic disease, regardless of protocol, were excluded, as well as patients with ECOG p.s. > 1. Both drugs were given by intravenous infusion on days 1, 8, and 15 in a 28-day cycle. Gemcitabine and nab-paclitaxel were administered at doses of 1000 and 125 mg/m^2^, respectively. Treatment pauses due to toxicity were allowed, as well as regimen de-escalation to a single drug which was not considered a second line. The patients continued the treatment protocol until radiological and/or clinical disease progression, or unacceptable toxicity. Blood samples were taken prior to each administration of chemotherapy in order to monitor for myelotoxicity with appropriate dose adjustments or treatment postponement, if necessary. Objective evaluation of response to therapy with computed tomography, carbohydrate antigen 19-9, complete blood count and biochemistry, was routinely performed every 3 cycles.

The objectives of this study were to compare the effectiveness in terms of OS and progression-free survival (PFS) with regard to standard first line chemotherapy protocols for mPDAC in the real-world setting. The follow-up was set at 36 months. OS was defined as time from the start of treatment until death, or the last follow-up visit, and PFS as time from the start of treatment until failure of the first-line treatment. Treatment failure was considered at the time of disease progression or death from any cause.

Apart from age and sex, other variables were also obtained, including ECOG p.s., prior resection of the primary tumor, metastatic burden, metastases to the liver and/or peritoneum. The Shapiro–Wilk test was used to assess the normality of distribution. Continuous variables (age) were reported as medians and the difference was tested using a Mann–Whitney *U* test. Categorical variables were reported as absolute numbers and percentages and the differences tested using Pearson’s chi-squared test. Survival (OS and PFS) was estimated using the Kaplan–Meier method and curves of the different treatment groups were compared using the log-rank test. Cox regression analysis was used to examine the association between survival, the treatment protocol, and other influencing factors. Data on OS and PFS were censored at 36 and 24 months cutoff, respectively. Results were expressed as hazard ratios (HR) with a 95% confidence interval (CI) with statistical significance set at a confidence level of *P* < .05.

## 3. Results

### 3.1. Patient and disease characteristics

The median age for the 139 patients included in the study was 67 years with patients in the Nab/P-Gem being significantly younger than the ones in the Gem arm (62.5 vs 69 years, *P* < .001) (Table 1). In the Gem group, 53 patients (70.7%) were older than 65 years and 22 (29.3%) were younger compared to 30 (46.9%) and 34 (53.1%) in the Nab/P-Gem arm, respectively. Apart from their older age, patients in Gem arm appeared to be in worse overall condition. In the Gem arm, 38 (50.7%) patients were ECOG p.s. 1 as opposed to 19 (29.7%) in the Nab-P/Gem arm, respectively. Significantly more patients had disease dissemination to the liver who received Nab-P/Gem (*P* = <.001). No significant difference existed between the 2 groups with regard to sex (*P* = .734); and in both treatment arms, the ratio of men to women was approximately 2:1. Furthermore, both arms were similar in terms of the number of patients having received prior surgery of the primary tumor (*P* = .263), metastatic burden (1–2 vs ≥3 organs affected with metastases; *P* = .055), and peritoneal dissemination of pancreatic cancer (*P* = .729).

**Table 1:** Patient characteristics at baseline (N = 139).

		All, N = 139	Nab-paclitaxel + Gemcitabine, N = 64	Gemcitabine, N = 75	*P*
Median age (yr)		67	62.5	69.0	<.001
		n (%)	n (%)	n (%)	
Sex					.734
	Women	63 (45.3)	30 (46.9)	33 (44.0)	
	Men	76 (54.7)	34 (53.1)	42 (56.0)	
Age distribution					.004
	<65 yr	56 (40.3)	34 (53.1)	22 (29.3)	
	≥65 yr	83 (59.7)	30 (46.9)	53 (70.7)	
ECOG performance status					.012
	0	82 (59.0)	45 (70.3)	37 (49.3)	
	1	57 (41.0)	19 (29.7)	38 (50.7)	
Prior surgery					.263
	Yes	39 (28.1)	15 (23.4)	24 (32.0)	
	No	100 (71.9)	49 (76.6)	51 (68.0)	
Metastatic burden (N. of sites)					.055
	1–2	106 (76.3)	44 (68.8)	62 (82.7)	
	≥3	33 (23.7)	20 (31.3)	13 (17.3)	
Metastatic site liver peritoneum					<.001
	Yes	94 (67.6)	53 (82.8)	41 (54.7)	
	No	45 (32.4)	11 (17.2)	34 (45.3)	
					.729
	Yes	35 (25.2)	17 (26.6)	18 (24.0)	
	No	104 (74.8)	47 (73.4)	57 (76.0)	

ECOG = Eastern Cooperative Oncology Group.

### 3.2. Survival analysis and treatment outcomes

We used the Cox regression model to ascertain the effects of age, sex, ECOG p.s., prior surgery of the primary tumor, metastatic burden, metastatic disease affecting the liver or peritoneum, and therapy protocol received on the OS and PFS as outcomes. In both survival analyses, the model showed statistical significance (Table 2). We did not find a difference in survival for the age and sex of the patients, as well as metastatic burden and metastases to the peritoneum. However, there was a significant difference in OS and PFS for ECOG p.s., so that the patients of a poorer general condition (ECOG p.s. 1) had a shorter survival independent of therapy (OS HR, 95% CI: 0.44, 1.34–3.71, *P* < .001; PFS HR, 95% CI: 0.50, 0.34–0.73, *P* < .001). Not having a prior surgery was negatively correlated with OS (HR, 95% CI: 2.23, 1.34–3.71, *P* = .002), but not PFS (HR, 95% CI: 1.37, 0.92–2.05, *P* = .123). Curative resection with negative margins where the tumor has not involved the vasculature is a favorable prognostic factor for long-term survival.^[18,19]^ Historically, <20% of patients with pancreatic cancer undergoes surgery with curative intent. In our studied population, almost 30% of patients had this type of procedure, although all ultimately developed stage 4 disease. The above may imply that our population generally had more unnecessary surgical interventions. The reason may exist in radiological understanding as we do not routinely use magnetic resonance imaging which has greater sensitivity in detection of small liver metastases than computed tomography.^[20]^ On the other hand, in patients who underwent surgery with curative intent, it is possible that they have a more indolent disease, given that this population was not radiologically in stage 4 at the time of diagnosis.

**Table 2:** Cox regression model for OS and PFS as outcomes.

Demographics and disease characteristics		OS		
		Median (95% CI)	HR (95% CI)	*P* value
Age distribution	<65 yr	10.33 (3.72–16.94)	1.15 (0.69–1.92)	.603
	≥65 yr	9.87 (6.75–13.00)	1.00	
Sex	Women	11.83 (4.21–19.46)	1.13 (0.74–1.75)	.572
	Men	9.37 (7.94–10.80)	1.00	
ECOG	0	15.80 (11.02–20.58)	0.44 (0.28–0.69)	<.001
	1	7.63 (11.02–20.58)	1.00	
Prior surgery	No	9.37 (7.15–11.59)	2.23 (1.34–3.71)	.002
	Yes	15.53 (8.90–22.16)	1.00	
Metastatic burden	1–2	9.17 (7.42–10.92)	1.73 (0.96–3.09)	.066
	≥3	3.28 (7.80–20.66)	1.00	
Liver	No	10.17 (2.68–17.67)	0.42 (0.25–0.70)	.001
	Yes	10.33 (7.78–12.89)	1.00	
Peritoneum	No	10.13 (7.69–12.57)	0.82 (0.48–1.41)	.474
	Yes	10.33 (10.21–17.45)	1.00	
Therapy	Abr-Gem	13.87 (8.14–19.60)	0.48 (0.29–0.78)	.003
	Gem	8.47 (6.56–10.38)	1.00	
Demographics and disease characteristics		PFS		
		Median (95% CI)	HR (95% CI)	*P* value
Age distribution	<65 yr	4.87 (2.93–6.81)	1.01 (0.66–1.55)	.956
	≥65 yr	3.20 (1.62–4.78)	1.00	
Sex	Women	3.20 (1.20–5.20)	0.93 (0.65–1.35)	.716
	Men	3.67 (2.10–5.24)	1.00	
ECOG	0	5.47 (4.43–6.51)	0.50 (0.34–0.73)	<.001
	1	2.63 (2.28–2.98)	1.00	
Prior surgery	No	3.50 (2.22–4.78)	1.37 (0.92–2.05)	.123
	Yes	4.83 (1.81–7.85)	1.00	
Metastatic burden	1–2	3.47 (2.59–4.36)	1.54 (0.95–2.50)	.080
	≥3	5.37 (3.34–7.40)	1.00	
Liver	No	3.80 (1.23–6.37)	0.64 (0.42–0.99)	.046
	Yes	3.50 (1.98–5.02)	1.00	
Peritoneum	No	3.53 (2.17–4.89)	0.91 (0.58–1.44)	.691
	Yes	4.40 (1.89–6.91)	1.00	
Therapy	Abr-Gem	5.37 (4.23–6.51)	0.71 (0.48–1.07)	.103
	Gem	2.80 (2.30–3.31)	1.00	

CI = confidence interval, ECOG = Eastern Cooperative Oncology Group, Gem = gemcitabine, HR = hazard ratio, OS = overall survival, PFS = progression-free survival.

**Figure 1.**
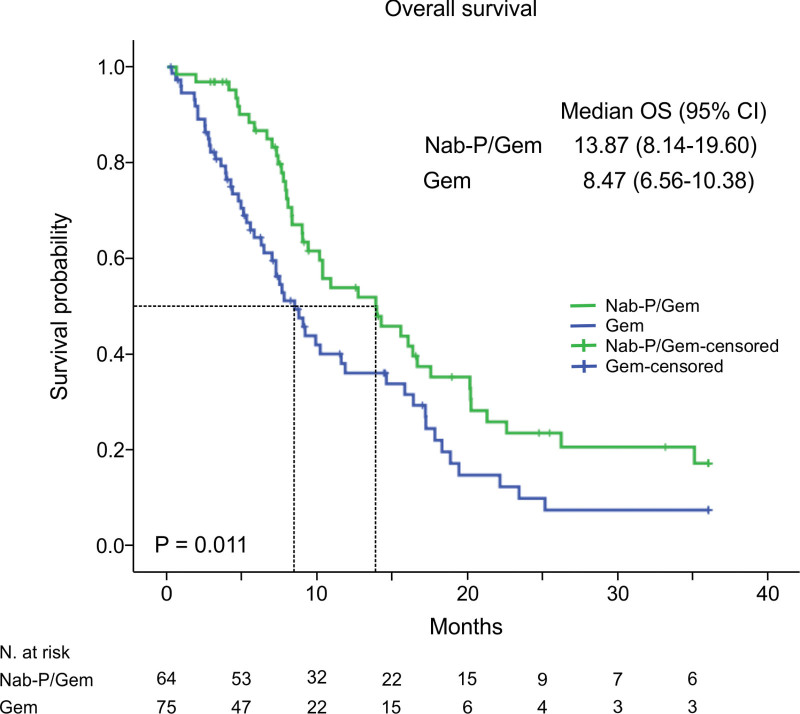
Kaplan–Meier estimates of overall survival (months). CI = confidence interval, HR = hazard ratio.

**Figure 2.**
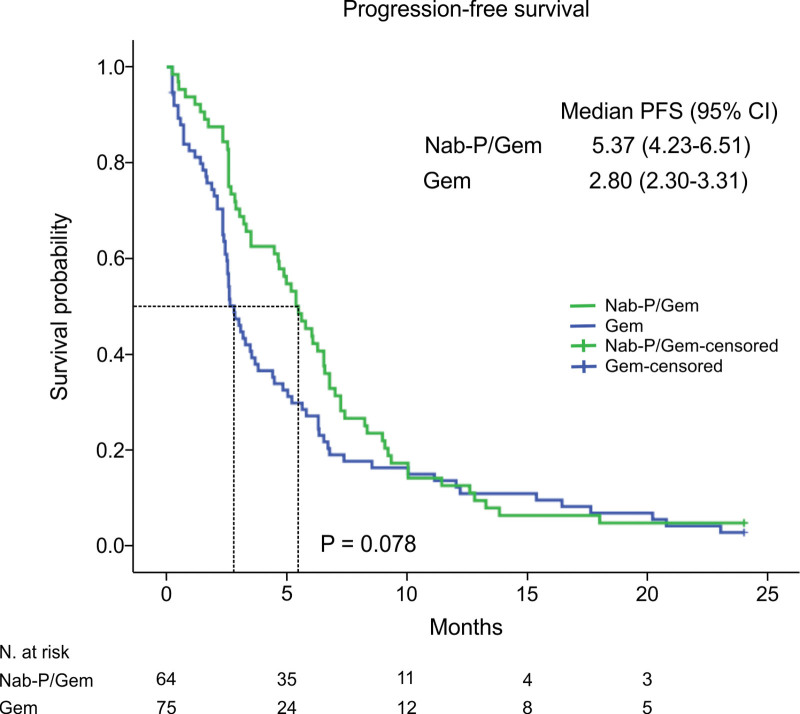
Kaplan–Meier estimates of progression-free survival (months). CI = confidence interval, HR = hazard ratio.

Similarly, patients with metastases to the liver had significantly shorter both OS and PFS (OS HR, 95% CI: 0.42, 0.25–0.70, *P* = .001; PFS HR, 95% CI: 0.64, 0.42–0.99, *P* = .046). Furthermore, patients who received Nab-P/Gem had statistically significantly longer OS than the patients who received Gem, 13.87 versus 8.47 months, respectively (HR, 95% CI: 0.48, 0.29–0.78, *P* = .003) with 6-month OS of 78.1% for Nab-P/Gem and 54.7% for Gem (*P* = .004) (Table 2,3) (Fig. 1). Consistent with this result, the same tendency in favor of Nab-P/Gem was demonstrated for PFS, 5.37 versus 2.80 months (Fig. 2). Although this was not statistically significant (HR, 95% CI: 0.71, 0.48–1.07, *P* = .103).

**Table 3:** Overall survival, progression-free survival, and responses in the first line.

	Nab-paclitaxel + Gemcitabine, n = 64		Gemcitabine monotherapy, n = 75			HR (95% CI)	*P* value
Overall survival median (95% CI)	13.87 (8.14–19.60)		8.47 (6.56–10.38)			0.48 (0.30–0.76)	.003
6 mo OS, N (%)	50 (78.1)		41 (54.7)			–	.004
	Nab-paclitaxel + Gemcitabine, n = 64		Gemcitabine monotherapy, n = 75			HR (95% CI)	*P* value
Progression-free survival, median (95% CI)	5.37 (4.23–6.51)		2.80 (2.30–3.31)			0.71 (0.48–1.05)	.103
Response in the first line	No. of patients (%)						*P* value
Complete response	0	(0.0)		0	(0.0)		–
Partial response	19	(29.7)		7	(9.3)		.004
Stable disease	19	(29.7)		26	(34.7)		.588
Progressive disease	21	(32.8)		35	(46.7)		.119
Could not be evaluated	5	(7.8)		7	(9.3)		1.000
Disease control rate	38	(59.4)		33	(44.0)		.089
Received second line	27	(42.2)		23	(30.7)		.334

CI = confidence interval, HR = hazard ratio, OS = overall survival.

**Figure 3.**
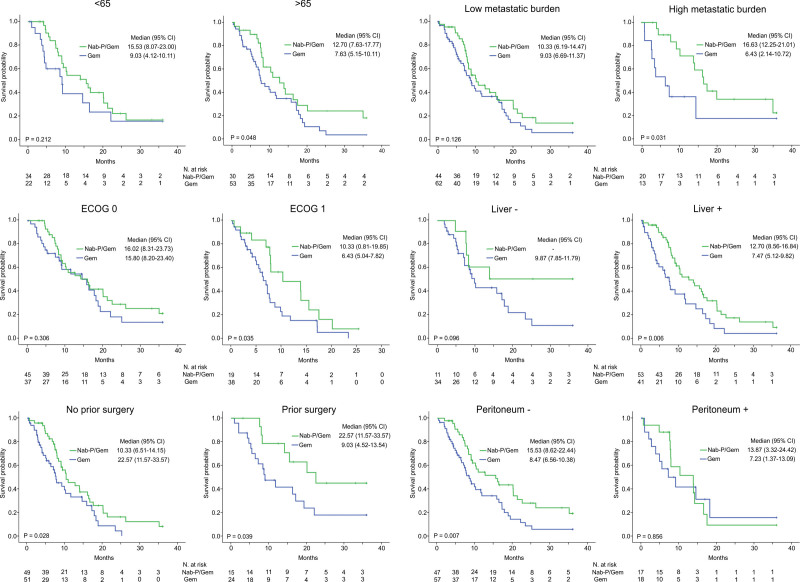
Kaplan–Meier estimates of overall survival (months) in specific subgroups. (A) Age distribution; (B) ECOG p.s.; (C) Prior surgery; (D) Metastatic burden; (E) Liver metastases; (F) Peritoneal metastases. CI = confidence interval, ECOG = eastern cooperative oncology group, HR = hazard ratio, p.s. = performance status.

The log-rank Mantel-Cox test was used for comparison of 2 treatment groups survival outcomes for each subgroup separately; including age, ECOG p.s., prior surgery of the primary tumor, metastatic burden, and metastases to the liver. A significant OS benefit of Nab-P/Gem versus Gem was found in the population older than 65 years (12.70 vs 7.63 months, *P* = .048) as well as for the patients of ECOG p.s. 1 (10.33 vs 6.43 months, *P* = .035), higher metastatic burden (16.63 vs 6.34 months, *P* = .031), and affection of the liver (12.70 vs 7.47 months, *P* = .006). In contrast, in younger patients, those of ECOG p.s. 0, lower metastatic burden (<3 organs affected), and those without metastases to the liver there was no difference in OS (Fig. 3a–f). In line with these results, significant PFS benefit was seen in patients who had ECOG p.s. 1 (4.47 vs 2.50 months, *P* = .041), higher metastatic burden (6.77 vs 2.83 months, *P* = .030), and liver metastases (5.37 vs 2.60 months, *P* = .012). There was no significant PFS difference in other subgroups (data not shown). OS benefit of Nab-P/Gem was achieved regardless of prior surgery, both for the patients with primary tumor in situ and the ones whose primary pancreatic cancer was resected, 10.33 versus 7.63 months (*P* = .028) and 22.57 versus 9.03 (*P* = .039), respectively.

Among patients in the Nab-P/Gem group, with regard to a smaller number of patients, a significant benefit in OS was observed among patients with higher metastatic burden (HR 4.11, *P* = .002), who received prior surgery (HR 3.02, *P* = .020), and no metastases to the liver (HR 0.304, *P* = .038). No significant influence on survival was found for age, ECOG p.s. or peritoneal dissemination (data not shown).

Using response evaluation criteria in solid tumors1.1 for a response evaluation, no complete response was observed to either treatment; significantly more patients achieved partial response in Nab-P/Gem than in the Gem group, 29.7 and 9.3% (*P* = .004), respectively (Table 3). However, this did not translate into a difference in the disease control rate. Overall, 59.4% of patients receiving Nab-P/Gem and 44% receiving Gem, achieved disease control rate (*P* = .089). In terms of response and eligibility for the second-line treatment, no other statistically significant difference was found although a numerically higher proportion of patients received second line in the Nab-P/Gem group (42.2% vs 30.7%).

## 4. Discussion

The first-line treatment of patients with mPDAC has significantly improved with more effective chemotherapy protocols available in recent years. We presented evidence on the effectiveness of Nab-P/Gem and Gem in our academic institution in a real-world setting. For data collection we also included a time frame in which monotherapy with gemcitabine was the only available option for mPDAC. This indicate how the addition of nab-paclitaxel to gemcitabine affects the mPDAC treatment outcomes in a single institution. Additionally, we demonstrated which patients benefited the most from the more intensive therapy.

We found a longer PFS with a median of 5.37 months in favor of Nab-P/Gem, although this was not statistically significant. The median PFS in the nab-P/Gem group was in line to the results of MPACT phase III trial of 5.5 months with overlapping confidence intervals (95% CI 4.5–5.9 month).^[7]^ On the other hand, mono Gem resulted in a much shorter PFS than nab-P/Gem therapy, worse than in the MPACT, 2.80 and 3.7 month, respectively. The statistical difference was achieved for the OS with Nab-P/Gem having a 5.4 month longer survival than Gem (HR 0.48, 95% CI 0.30–0.76). In our study both arms separately achieved longer OS compared to the MPACT trial in which OS for Nab-P/Gem and Gem were 8.5 and 6.7 months, respectively.^[7]^

Notably, there are some differences in our study population when comparing it to the MPACT trial. Our patients were generally older, most being older than 65 years and had prior surgery. Additionally, the majority of our patients had <3 radiologically confirmed metastatic sites. Furthermore, given the nature of the study design, and the aforementioned characteristics of the study population, direct comparison is not feasible. With different outcomes depending on the therapy, prognostic factors such as advanced ECOG p.s., prior pancreatic cancer surgery, and liver involvement were associated with a shorter survival. Taking into account our results with those in the MPACT trial, we can say that patients with advanced age, poor p.s. (ECOG p.s. 1), presence of liver metastasis, or more than 3 metastatic sites tend to have worse survival outcomes.^[21]^

The subgroup analysis, interestingly, showed that older patients, patients with worse p.s. (ECOG p.s. 1 as opposed to 0), and with greater metastatic burden, benefited the most from nab-P/Gem in terms of OS. Apart from age, these 2 factors were also positive predictors for PFS in nab-P/Gem arm. In contrast to OS, no difference was seen in PFS in patients older than 65 years. Similarly, no difference in median PFS was recently reported in a non-interventional study by Prager et al^[22]^ where patients receiving nab-P/Gem were stratified by age. The median PFS was 5.55 and 5.52 months for patients ≤70 and >70, respectively. They found no difference in OS between the 2 arms with a median 10.6 in younger and 10.2 months in older patients. One other study involving 78 patients by Macchini et al^[23]^ also found that both single agent treatment and Nab-P/Gem resulted in similar efficacy in patients over and under 75 years. However, monochemotherapy (arm A) resulted in worse OS compared to Nab-P/Gem (arm B), 7.9 versus 11.7 months, respectively.

Regarding p.s., we treated more patients with worse ECOG p.s. with gemcitabine monotherapy than with Nab-P/Gem (Table 1). Unfortunately, gemcitabine alone has very limited value in mPDAC treatment^[13,22,24]^ and the patients’ general condition is recognized as an independent negative predictive factor for survival.^[14,15,21]^ With that in mind, a more intensive therapeutic approach appears to offer greater survival benefits in patients with more advanced p.s. (ECOG p.s. 1 as opposed to 0) which is often in contrast to our current clinical practice.

Survival results for the Nab-P/Gem group in our study are consistent with other reported real-world data; in 2 single-arm studies DeVita et al^[16]^ reported 10 months OS and 6.7 months PFS while Blomstrand et al reported 9.4 and 4.5 months for mPDAC OS and PFS, respectively.^[17]^ A phase II study by Macarulla T et al^[25]^ investigated different dosing regimens of nab-P in arms B (100 mg/m^2^) and D (125 mg/m^2^). The authors reported similar OS results of 7.7 and 9.8 months and PFS of 5.7 and 6.7. In the mentioned trials the studied population was predominantly older with median age being over 65 years. The OS in these trials is comparable with median OS of 12.7 months in patients over 65 years who received Nab-P/Gem in our studied population.

Only a few studies addressed the issue of number and specific metastatic sites on prognosis of mPDAC.^[26,27]^ Meta-analysis by Usón^[27]^ regarding number of metastatic sites (metastatic burden), combining 2 phase III studies,^[6,7]^ found no impact on OS. The impact of peritoneal carcinomatosis is unknown due to the lack of reported data, although the results from MPACT trial^[21]^ suggest the addition of Nab-P was beneficial only when carcinomatosis was absent. The authors interpret these results with caution given the small number of patients and unbalanced groups. However, unlike the number of metastatic sites which has no impact on OS, liver involvement proved to be a poor prognostic sign both in terms of OS and PFS.^[27]^ Furthermore, the liver involvement appears to be a predictive factor as well. In fact, patients with pancreatic cancer and liver metastases benefit more from nab-P/Gem than Gem alone, although liver mets are associated with worse prognosis. Our results indicate that these patients benefit from more intensive chemotherapy in terms of both PFS and OS. Similarly, in a study by Macchini et al,^[23]^ the difference in OS was more pronounced when patients with only lung metastases, who comprised <18% of the total cohort, were excluded. In contrast, liver metastases were present in over 68% of patients. Most patients in our study (82.8%) who received Nab-P/Gem had liver metastases as opposed to 54.7% in the Gem group.

Our study has limitations primarily related to its retrospective design. It is conducted in a single institution with bias regarding treatment choice by the attending oncologist. Furthermore, the interpretation of the subgroup analysis is limited to its relatively small sample size. The heterogeneity of therapies applied in later treatment lines is also a possible factor influencing the OS outcome. Unfortunately, less than half of the patients in either group were eligible to receive subsequent therapy. The optimal sequence of treatment and the effect of choosing a second line remains to be explored.

In conclusion our results provide additional data on the effectiveness of Nab-P/Gem in mPDAC in the real single-institution setting. The OS benefit is comparable with the results from previous observational studies, and greater when compared to MPACT trial. Furthermore, our results suggest that Nab-P/Gem is effective in patients with unfavorable prognostic factors, for example, age >70, ECOG p.s. 1, higher metastatic burden, and liver involvement.

## Acknowledgments

The authors thank the University Hospital Centre Zagreb for providing the data for the research. We are also greatly indebted to Prof M. Sirotković Skerlev, MD, PhD for critically reviewing the manuscript, Mr. Dalibor Koštan for his help with English language editing of the manuscript, and Dr Tamara Poljičanin, MD, PhD for her help with statistical analysis.

## Author contributions

**Conceptualization:** Juraj Prejac.

**Data curation:** Juraj Prejac, Dora Tomek Hamzić, Domina Kekez.

**Methodology:** Juraj Prejac.

**Project administration:** Stjepko Pleština.

**Supervision:** Juraj Prejac, Nikša Librenjak, Irma Goršić.

**Validation:** Irma Goršić.

**Visualization:** Stjepko Pleština.

**Writing – original draft:** Juraj Prejac, Dora Tomek Hamzić, Nikša Librenjak.

**Writing – review & editing:** Irma Goršić, Domina Kekez, Stjepko Pleština.
